# Secukinumab-Induced Crohn's Disease in a Patient Treated for Juvenile Idiopathic Arthritis

**DOI:** 10.7759/cureus.43825

**Published:** 2023-08-20

**Authors:** Srujan Edupuganti, Su Khine, Rohit Gupta, Deepesh Yadav, Adiraj Singh.

**Affiliations:** 1 Internal Medicine/Pediatrics, Michigan State University College of Human Medicine, Hurley Medical Center, Flint, USA; 2 Internal Medicine, Michigan State University College of Human Medicine, Hurley Medical Center, Flint, USA; 3 Rheumatology, University of Arkansas for Medical Sciences, Little Rock, USA; 4 Orthopedic Surgery, Kathmandu University, Dhulikhel, NPL

**Keywords:** il-17a, interleukin-17a blockers, il-17, interleukin-17, ibd, inflammatory bowel disease, crohn's disease, jia, juvenile idiopathic arthritis, secukinumab

## Abstract

Juvenile idiopathic arthritis (JIA) is a common form of arthritis that occurs in children, typically with an onset before the age of 16 years. It can affect joints in any part of the body. As per the International League of Rheumatology, JIA is classified into systemic arthritis, oligoarthritis, extended oligoarthritis, polyarthritis (rheumatoid factor positive), polyarthritis (rheumatoid factor negative), enthesitis-related arthritis (ERA), juvenile psoriatic arthritis (JPsA), and *other arthritis*. JIA is treated with disease-modifying antirheumatic medications (DMARDs), which include both nonbiologic agents like methotrexate (MTX) and biologic agents like inhibitors of tumor necrosis factor-alpha, interleukin-1 (IL-1), IL-6, and T-cell co-stimulation modulators. As per recent studies, in December 2021, Secukinumab, an IL-17A inhibitor, is one of the most recent biologic agents approved for active ERA and JPsA. A few reports have suggested Secukinumab is related to new-onset inflammatory bowel diseases (IBDs). We present a case of a 20-year-old female who was being treated with Secukinumab for JIA, and six months into therapy, she developed symptoms suggestive of Crohn’s disease (CD). The diagnosis was confirmed with colonoscopy, histopathology, and radiology results. Her symptoms completely resolved four weeks after discontinuing Secukinumab and oral steroid therapy. The efficacy and side effects of Secukinumab have been studied mainly on middle-aged populations who were being treated for psoriasis and ankylosing spondylitis (AS); however, there is limited literature on younger populations. With this case report, we would like to highlight the possible relationship between the development of IBD and Secukinumab therapy in the adolescent population and emphasize the importance of regular screening for IBD in this population.

## Introduction

Juvenile idiopathic arthritis (JIA) is a chronic idiopathic inflammatory disorder that mainly affects joints, with disease onset before the age of 16 and symptoms lasting more than six weeks. As per the International League of Rheumatology, JIA is classified into systemic arthritis, oligoarthritis, extended oligoarthritis, polyarthritis [rheumatoid factor (RF) positive], polyarthritis (RF negative), enthesitis-related arthritis (ERA), juvenile psoriatic arthritis (JPsA), and *other arthritis*. The prevalence of JIA among children and adolescents is estimated to be around 3,000,000 globally by 2021 [1]. African American children are more likely to have a polyarticular form unlike Caucasian and Japanese children, who most often have an oligoarticular [2] and systemic forms, respectively [3]. Treatment options include nonbiologic agents like methotrexate (MTX) and biologic agents like inhibitors of tumor necrosis factor-alpha (TNF-alpha), interleukin 1 (IL-1), IL-6, and T-cell co-stimulation modulators [4]. In December 2021, a recent biologic agent Secukinumab, an IL-17A inhibitor, was licensed for the treatment of active ERA, and active JPsA. Although uncommon, new-onset inflammatory bowel diseases (IBDs) were reported in patients treated with Secukinumab, but most of the studies are focused on middle-aged people being treated for psoriasis (PsO), psoriatic arthritis (PsA), and ankylosing spondylitis (AS) [5]. We are presenting a case of a 20-year-old female with a history of JIA who was diagnosed with Crohn’s disease (CD) six months after initiation of Secukinumab.

## Case presentation

**Figure 1:**
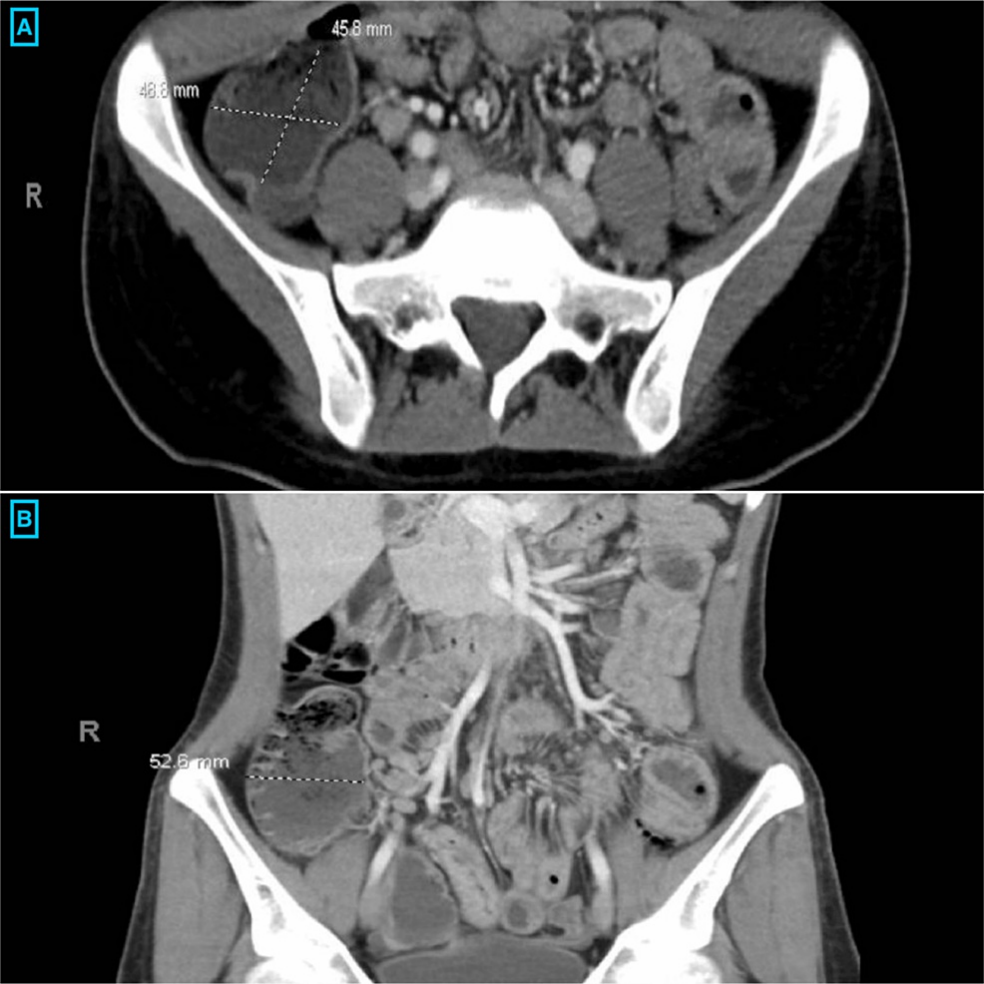
CT of the abdomen and pelvis: terminal ileitis, evidenced by an enlarged bowel wall in the (A) axial view and (B) coronal view. CT, computerized tomography

**Figure 2:**
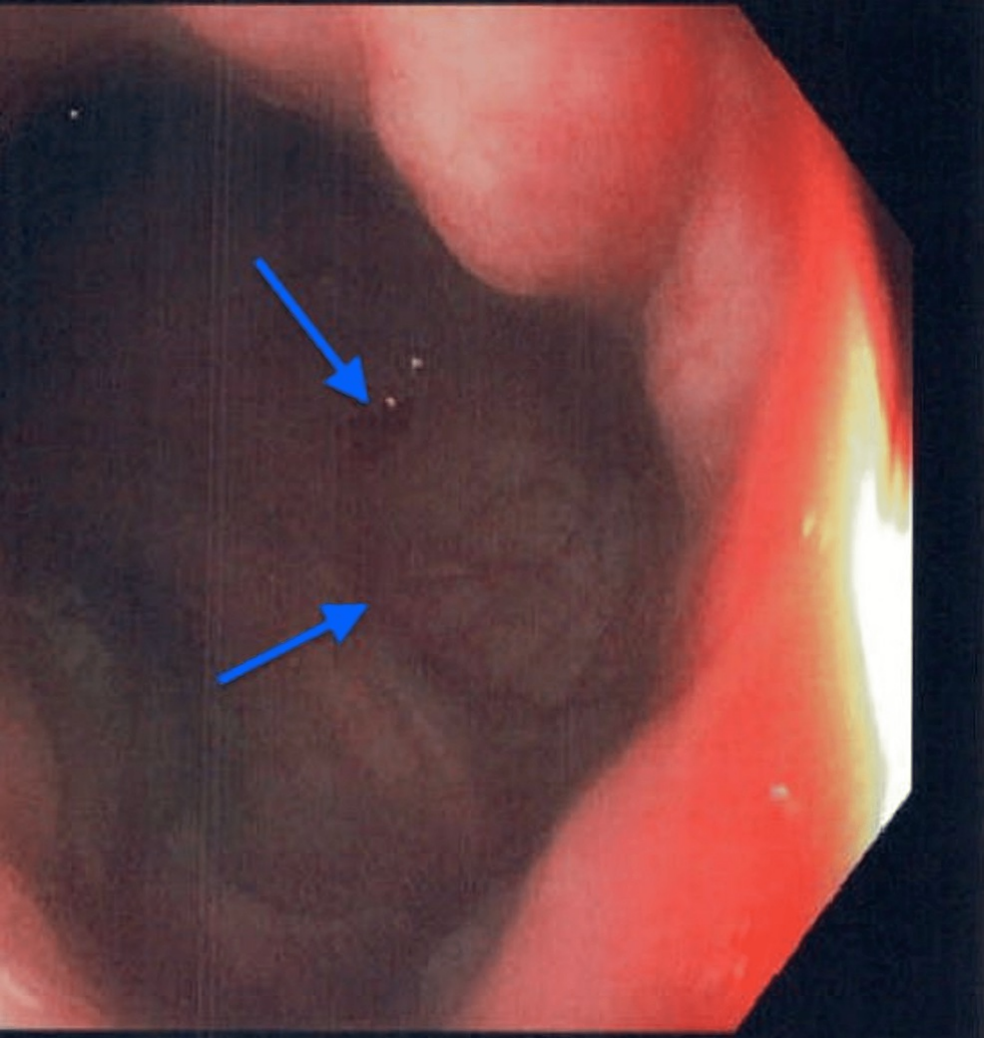
Colonoscopy showing terminal ileitis as indicated by the arrows.

**Figure 3:**
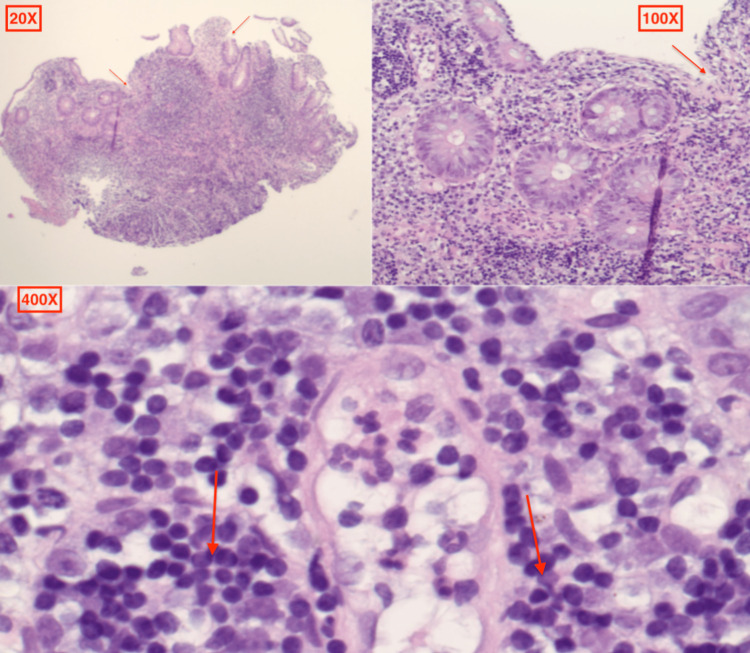
H&E photomicrograph of ileal mucosa revealing surface ulceration with acute and chronic inflammatory changes, as indicated by the arrows. H&E, hematoxylin and eosin

The patient was a 20-year-old Caucasian female who presented to the emergency department (ED) with complaints of abdominal pain, non-bloody diarrhea, and vomiting that had been occurring for eight days. She reported over 20 episodes of non-bloody bowel movements per day, associated with non-bloody, non-bilious emesis and fever with a maximum temperature of 101 ℉ (38.3 ℃). Her past medical history was significant for a five-year history of radiographically proven, antinuclear antibody (ANA) positive (1:160) JIA/spondyloarthropathy with involvement of temporomandibular joint (TMJ). She had failed MTX and Infliximab. Hence, seven months ago, she started on Secukinumab 300 mg monthly injections and Mycophenolate. On initial evaluation in the ED, she was afebrile but was tachycardic with a heart rate of 165 beats per minute. She was clinically dehydrated with generalized abdominal tenderness, more pronounced in the right lower quadrant. Laboratory results were remarkable for electrolyte disturbances with elevated erythrocyte sedimentation rate (ESR) and C-reactive protein (CRP) levels, as shown in Table 1. The fecal occult blood test was positive. Stool studies for ova and parasites, adenovirus, and *Clostridium difficile* toxin assay were negative. Computerized tomography (CT) of the abdomen and pelvis revealed small bowel wall thickening most significantly involving the terminal ileum with associated vasa recta engorgement, suggestive of CD (Figure 1). Rheumatology and gastroenterology were consulted. Colonoscopy revealed terminal ileitis characterized by erythema, congestion, and ulceration with the normal gross appearance of the colon (Figure 2). Multiple biopsies were taken from the ileum and sent for histopathology, which revealed small intestinal mucosa containing surface ulceration with underlying acute and chronic inflammation without any granulomatous changes (Figure 3). After correlating the patient's presentation, and imaging, with gross and histopathology findings from the colonoscopy, CD was diagnosed. Given recent studies associating Secukinumab with the new-onset IBD, the medication was discontinued. The patient was started on intravenous Methylprednisolone 30 mg twice a day. She was later transitioned to oral prednisone with a tapering dose. Subsequently, Upadacitinib was initiated after bridging with steroids for cross-coverage of both CD and JIA. Her symptoms completely resolved after four weeks following the discontinuation of Secukinumab.

**Table 1:** Relevant laboratory values. ESR, erythrocyte sedimentation rate; CRP, C-reactive protein

Blood test	Results
Sodium	129 mEq/L
Potassium	3.5 mEq/L (1+hemolysis)
Chloride	92 mEq/L
ESR	23 mmHg/hour
CRP	269.94 mg/L

## Discussion

JIA has been related to increased risks of developing IBD in several studies. A study showed that the incidence of IBD is higher in patients with JIA than in the general pediatric population [6]. In another study that involved 8,942 patients with JIA, it was revealed that patients with ERA or with a family history of autoimmune disease are at higher risk of developing IBD. It was determined that patients with JIA on Etanercept and Infliximab had a greater risk of developing IBD, regardless of MTX use. The study concluded by recommending the consideration of adalimumab (TNF-alpha inhibitor), especially for ERA patients with a family history of autoimmune disease [7]. MTX, on the other hand, has been reported to have a protective effect against IBD in patients with JIA [8]. However, there hasn't been much research done on the incidence of IBD in individuals undergoing treatment with other biologic medications for JIA.

Secukinumab is a novel biologic agent that was initially approved for PsA and AS in 2016. It was recently approved for the treatment of ERA and JPsA in pediatric patients in the United States due to promising results of the JUNIPERA trial [9]. The trial demonstrated a significantly extended time to flare in the Secukinumab group, maintaining a consistent safety profile within the younger population as compared to the adult cohorts. However, the trial did report a case in which a new-onset IBD developed, necessitating the discontinuation of Secukinumab treatment. The trial also recognized a possible causal relationship between IBD and Secukinumab, but we do not have enough data among the pediatric population signifying this relationship. However, there are some reports where adult patients developed new-onset IBD or had worsening preexisting IBD while being treated with Secukinumab for plaque PsO and PsA [10,11,12]. One suggested mechanism was that neutralization of IL-17A resulted in the deterioration of the intestinal epithelial barrier exacerbating intestinal inflammation [13]. Further studies should be conducted on underlying pathophysiology, and more guidelines should be set for the use of IL-17 inhibitors in patients with high risk for IBD.

In addition, pediatric populations are at higher risk for developing a more severe and extensive type of IBD, which is related to growth failure, malnutrition, and increased fracture risks [14]. They are also at increased risk of long-term consequences such as intestinal strictures, obstruction, fistula, and cancer. Physicians need to recognize the symptoms of IBD early in such populations. A thorough gastrointestinal history and physical exam should be performed before the initiation of Secukinumab or any other IL-17 blocker. Fecal calprotectin is also a screening test that could be considered to identify IBD in patients with JIA [15].

## Conclusions

In conclusion, it is advisable to assess patients with JIA for the presence of IBD before initiating therapy with Secukinumab. Additionally, maintaining a vigilant approach to IBD screening, both before and after the initiation of therapy, is recommended. Physicians need to identify IBD at an early stage in these vulnerable populations and consider halting Secukinumab if IBD is suspected. The onset of IBD screening and the interval of screening remain unclear and require clearer definitions. 
